# In Vitro Symbiotic Germination: A Revitalized Heuristic Approach for Orchid Species Conservation

**DOI:** 10.3390/plants9121742

**Published:** 2020-12-09

**Authors:** Galih Chersy Pujasatria, Chihiro Miura, Hironori Kaminaka

**Affiliations:** 1 Department of Agricultural Science, Graduate School of Sustainable Science, Tottori University, Tottori 680-8553, Japan; galihchersyp@gmail.com; 2 Faculty of Agriculture, Tottori University, 4-101 Koyama Minami, Tottori 680-8553, Japan; cmiura@tottori-u.ac.jp

**Keywords:** conservation, in vitro, mycorrhizal fungus, mycorrhizal symbiosis, orchid, seed germination

## Abstract

As one of the largest families of flowering plants, Orchidaceae is well-known for its high diversity and complex life cycles. Interestingly, such exquisite plants originate from minute seeds, going through challenges to germinate and establish in nature. Alternatively, orchid utilization as an economically important plant gradually decreases its natural population, therefore, driving the need for conservation. As with any conservation attempts, broad knowledge is required, including the species’ interaction with other organisms. All orchids establish mycorrhizal symbiosis with certain lineages of fungi to germinate naturally. Since the whole in situ study is considerably complex, in vitro symbiotic germination study is a promising alternative. It serves as a tool for extensive studies at morphophysiological and molecular levels. In addition, it provides insights before reintroduction into its natural habitat. Here we reviewed how mycorrhiza contributes to orchid lifecycles, methods to conduct in vitro study, and how it can be utilized for conservation needs.

## 1. Introduction

Orchidaceae is one of the biggest families of angiosperms with more than 17,000–35,000 members [1,2,3], including the famous *Phalaenopsis, Dendrobium,* and *Cattleya.* These orchids survive in almost every climate and ecosystem, ranging from sea level to tropical and temperate mountains [4,5,6]. Orchids exhibit terrestrial and epiphytic growth habits, and all produce numerous minute seeds that must establish symbiosis with certain fungi, referred to hereafter as orchid mycorrhizal fungi (OMF), which infrequently occurs under natural conditions [7].

Due to low regeneration rates caused by its difficulty to germinate and human interference, such as overcollection to fulfill economic and horticultural demands, the endangered status of many orchids remains a problem even today [8,9]. Globally, orchid conservationists attempt to balance market demands and biodiversity; thus, large-scale production is required. Commercially, there are several promising alternatives, such as tissue culture/cloning and in vitro asymbiotic germination [10,11]. However, both methods have disadvantages. Tissue culture/cloning offers identical characteristics with the mother plant, but clones may exhibit somaclonal variation. Such variation is usually undesirable due to its genetic instability and pleiotropic effects. Somaclonal variation is typically avoided in nature because it disrupts gene flow [12]. In addition, in vitro asymbiotic germination seems to overly indulge seedlings with readily available nutrients and ideal growing conditions. This is a problem if the seedlings are subjected to acclimatization in the wild where plantlets may face climatic variation, herbivory, and pathogens, which were completely absent during in vitro culture. Symbiotic culture allows seeds to interact with fungi from early developmental stages, which resembles natural conditions and results in better seedling growth [10,13]. Symbiotic culture also proved to be beneficial during greenhouse acclimatization [14]. Therefore, symbiotic germination is a promising alternative. To conserve orchids, which strongly rely on mycorrhizal symbiosis, conservation of the plant and knowledge about mycorrhizal fungi are required [15,16].

By studying fungal species, the entire mechanism of orchid mycorrhizal (OM) symbiosis can be explored. In addition, recent developments in molecular biology and advanced tools, such as isotope tracking, facilitate analysis of OMF diversity from natural habitat, omics, and isotope tracking. In this review, we provide insights into the biology of orchid seed germination, methods for conducting in vitro symbiotic germination, as well as its new advances, and its prospects for the conservation of endangered orchids. For reference, we highlighted some studies showing promising results for symbiotic germination.

## 2. Through the Looking Glass: How Orchids Naturally Establish

### 2.1. Orchid Seed Characteristics as Innate Strategies of Reproduction

As with most flowering plants, seed production is the primary strategy for generative reproduction in orchids. Orchids produce copious minute (0.34–24 µg, 0.01–0.9 × 0.05–6 mm) seeds consisting of a seed coat and rudimentary embryo, but lacking endosperm that is normally required as an initial energy resource during germination [17]. Orchids generally display low fruit set but high fecundity; one dehiscent capsule contains hundreds to millions of seeds. Such abundant seed production is the strategy for establishment in natural habitats since, in many cases, not all seeds can easily germinate under suitable conditions. Seed size also varies among different species. For example, *Epipogium aphyllum* and *Bletilla striata* produce seeds consisting of 8 and 700 cells, respectively [18]. A rare case occurs in *Thecostele alata*, which has 3–12 embryos per seed [19]. These unique traits facilitate extensive dispersal, especially via anemochory [19]. Terrestrial orchids, especially those growing in the understory, are less reliant on wind for seed dispersal because they do not necessarily require a higher location (e.g., tree bark) for seedling establishment. It was also revealed that not all orchids rely on anemochory. Instead, the seeds are dispersed through droppings of frugivorous crickets, as seen in *Apostasia nipponica* [2].

The only way for orchid seeds to germinate is by obtaining nutrients from an external source. In nature, this is achieved by mycorrhizal symbiosis, which is discussed later in this review. Germination usually takes several months to a year [20] since germinating orchid seeds do not directly produce leaf and root primordia. Instead, a protocorm develops during the germination process. Briefly, the protocorm resembles a mass of cells bigger than an embryo. It is often described as a globular, undifferentiated cell mass, which later forms rhizoid and leaf primordium. Its cells are also actively utilizing nutrients and dividing [18].

### 2.2. Environment and Mycorrhizal Fungi as Constraints to Orchid Survival

Terrestrial and epiphytic orchids are exposed to unfavorable conditions during their lifetime. Terrestrial orchids are often found in the topsoil, which may be rich in nutrients and microbiome diversity [21]. The edaphic environment is more likely to retain water and minerals better than the arboreal environment, which is favorable for seed germination. In addition, the underground ecosystem is home to diverse microbiota. Due to this richness, terrestrial orchid seeds are exposed to several other organisms with various behaviors, such as parasites, herbivores, and saprobes. Alternatively, the arboreal environment is drier, prone to desiccation, and exposed to weather changes. The ecological niche of epiphytic orchids in the forest canopy provides both inter- and intraspecific relationships within this ecosystem. Thus, epiphytic orchids are sensitive to the microsite and anthropogenic disturbances [22]. Epiphytic orchids evolved later and developed the ability to grow on the surface of substrates, such as tree bark, rock crevices, or even inorganic materials. Geographically, epiphytic orchids are more distributed in tropical than temperate regions due to higher humidity requirements. Epiphytic orchids fulfilled the following conditions: the ability to reach the host tree and to survive under conditions of drought or desiccation during its establishment [23], which is almost impossible for terrestrial orchids to survive in. Among these epiphytes, there is a small group, which occurs on smaller portions or extremities of tree branches with high irradiation, lower humidity, and lower mineral accumulation or both, called twig epiphytes. Some examples include *Pleurothallis, Angraecum,* and the equitant *Oncidium* alliance, which are mostly found in rainforests with high humidity [23,24,25,26].

Orchids seem to have strict requirements for their establishment, even if resources are already fulfilled. For example, even though a viable seed lands on a suitable substrate—with sufficient nutrients such as carbon and nitrogen compounds—it cannot easily absorb those nutrients. Like any microbiome, fungi with different nutritional strategies can be found living together in the same substrate. It is still unclear whether such fungi compete to invade orchid seeds or rather recognize chemical signals produced by orchid seeds [27]. In addition, it is unknown if orchid seeds secrete chemical signals to attract fungi for establishing mycorrhizal symbiosis like those of arbuscular mycorrhiza (AM) [28]. Therefore, if the seeds cannot establish mycorrhizal symbiosis with a fungus, it is most likely unable to germinate, or at least undergo early morphological changes.

In addition, orchids do not simply establish symbiosis with any fungus; hence, germination can only occur when a suitable fungus is present. Most recognized OMF genera are basidiomycetous fungi with an anamorphic stage resembling the well-known plant pathogen, *Rhizoctonia*, and are named “*Rhizoctonia*-like fungi” [26,29]. Though uncommon, studies showing non-*Rhizoctonia*-like fungi forming OM have increased since the early 2000s [20,30,31,32,33,34,35,36,37]. Those genera include strong pathogen [6,30,33,38], saprobes [39,40], ectomycorrhizal fungi [41,42], and ascomycetous fungi [20,43]. Another unique orchid trait is mycoheterotrophy, which involves the utilization of OM symbiosis to obtain carbon, but does not reciprocally provide the fungi any nutrients [42,44]. It can simply be said that this is a kind of “fungal parasitism”. Mycoheterotrophy occurs in different proportions. Most photosynthetic orchids undergo mycoheterotrophy during germination, while others evolve into being partially or fully mycoheterotrophic. Partial or full mycoheterotrophy is usually characterized by plants expressing rudimentary leaves or being leafless [42,45]. Due to sole dependence on OMF, mycoheterotrophic orchids are more prone to disturbances than their photosynthetic counterparts.

The lack of endosperm in orchid seeds drives its adaptation to the establishment of mycorrhizal symbiosis to obtain nutrients. Symbiosis starts at the early stage of germination. What makes OMF different from other mycorrhiza is that the hyphae form a peloton, which is a hyphal coil inside the host cell where nutrient exchange occurs [46,47]. Hyphae enter from the basal part of the protocorm, and colonization normally only occurs there. Pelotons are not permanently formed; instead, these are digested by protocorm cells, and concomitantly, nutrients from hyphae are released [18,46,48]. Because the duration of germination is relatively long, peloton formation and digestion should be continuous to provide adequate nutrients until the protocorm is able to photosynthesize. As a result, pelotons are often formed repeatedly in the same cell [14].

### 2.3. In Vitro Orchid Symbiotic Germination as a Tool for Studying Mycorrhizal Symbiosis

**Figure 1:**
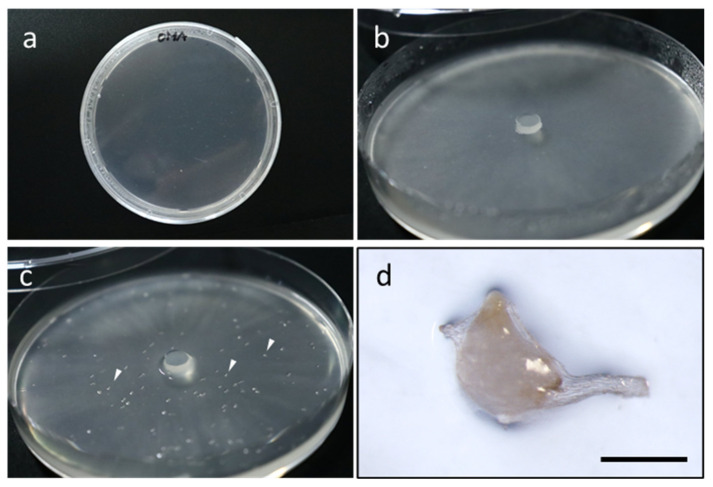
Preparation of in vitro symbiotic germination assay. (**a**) A plain oatmeal agar (OMA) plate. (**b**) A piece of fungal inoculum is put at the center of the plate. Hyphae will appear as a thin, whitish layer on the medium. (**c**) Surface-sterilized seeds (arrowhead) sown on top of the hyphal layer. (**d**) Symbiotically germinating *Bletilla striata* seed during inoculation with *Tulasnella* sp. after two weeks. Scale bars, 500 µm.

Although *in vitro* studies do not always represent realistic environmental conditions, these act as a microenvironment replica and may function as a tool for extensive studies [47,49]. The most convenient way to conduct in vitro study is by simply inoculating seeds and a suitable fungus on a solid agar medium (Figure 1a–c). This study requires two elements: (1) putative fungal symbiont and (2) a medium containing a nutrient source that is available to the fungus but not the orchid seed by assuming that the seeds can only absorb water, not nutrients. Although any medium suitable for growing fungus can be used, most studies utilized materials derived from natural sources, which lack inorganic salts, such as the commercially available oatmeal agar (OMA). Other less common media that only contain inorganic salts (e.g., Pfeffer’s and B&G salt mixture) are also suitable for several species [13,50]. When necessary, macronutrients [51] or trace elements can be added to observe the nutrient flow and their effect on protocorm-fungus interaction during symbiosis. To study the specificity of the fungus tested, OMF isolated from another species can be used. Several modifications for personal convenience, such as the usage of filter paper for seed support, can also be applied [52,53,54]. In some cases, the fungus is not necessarily in direct contact with the orchid seeds. In a study on *Cyrtosia septentrionalis*, a small box chamber was used for inoculation with no direct contact between the fungus and seeds [33]. Depending on the species, seed viability, OMF compatibility, or medium components, seeds can germinate in a month (Figure 1d) or less [48] or even up to a year [20,55].

According to Table 1, many orchids are considered generalist—or anecdotally, promiscuous—forming OM with fungi isolated from another orchid species [6,48,56,57,58,59,60]. For specialists, there are no other options but to use OMF isolated from respective plants [61]. Fungi can be obtained through peloton extraction from adult plant roots or protocorms grown in situ using the baiting method [8,62,63,64,65], isolation from plant disease symptoms [56], or isolation from the rhizosphere [54,66], as shown in Table 1. Each method has its pros and cons. Seed baiting mimics natural conditions where seeds directly interact with the substrate along with abundantly available fungi. In this method, the orchid seeds are inserted into small packs made of mesh with minute pores that still allow the entrance of fungal hyphae. The packs are placed on in situ plant growing substrate, often in proximity to the adult plant. For terrestrial orchids, the packs are simply buried, while in the case of epiphytes, the packs are mounted onto tree barks or placed inside an extensive root network [62]. Unfortunately, baiting often takes months or years, considering the ideal biotic and abiotic conditions for seeds to germinate. Often, the ratio of germinating seeds is low [8,12]. Compared to seedlings and adult plants, in situ protocorms are much harder to locate and usually require an expert [67]. For epiphytic orchids, protocorms are still possible to find because it is most likely fully exposed, but nearly impossible for terrestrial orchids because they are hidden and resembles soil particles. To compensate for this, the roots of adult plants are commonly used for fungus isolation by cutting a small section putatively containing pelotons and placing it on a culture medium [68,69,70,71]. Precautions need to be taken when dealing with orchids that switch fungal partners [11,72]. For such orchids, it is imperative to extract pelotons only from protocorms to avoid bias in germination assays.

When using morphological aspects as parameters, this caveat should be considered: rhizoid formation is commonly used as an assumption for the starting point of germination, but it is inaccurate since longer incubation time can alter early assumptions of germination results. For example, in a novel study for the determination of fungal symbiont, the fungus, which causes protocorms to form rhizoids earlier than other treatments, may be putatively regarded as a true mycobiont. However, with a longer incubation time, another fungus can promote better growth than the initial symbiont [68]. In addition, not all orchids are colonized by OMF after forming rhizoids; some OMF enter protocorms directly through epidermal cells instead of rhizoids [46]. Some species are also very easy to germinate; they germinate in the absence of OMF, even when sown in a symbiotic medium. The rest of the germination assay, such as seed morphological changes, germination percentage, duration of germination and development of protocorms, and growth index, can be conducted like that of asymbiotic germination [14,26,34,51,60,69].

## 3. New Knowledge Obtained from Symbiotic Germination Using Advanced Tools

Successfully germinated protocorms containing pelotons usually show no conspicuous symptoms, although they occasionally collapse due to unsuitable temperatures and nutrient sources [73,74], leading to disputes whether OM symbiosis is mutualistic or antagonistic [75]. Recent studies have approached this topic from a molecular level using the in vitro symbiotic germination method (Table 2). Pyrosequencing was used to explore transcripts of symbiotically germinated protocorms, and no significant induction of pathogenesis and wound/stress-related genes were observed [75]. Although plant defense-related proteins were identified during the symbiotic germination process of *Oncidium sphacelatum* and *Dendrobium officinale*, some responses shared at least a part of AM symbiosis, which involves a mutually beneficial interaction [44,76]. Another study using *B. striata* showed some common AM symbiosis features at the gene ontology level, suggesting that orchids possess, at least partly, the molecular mechanisms common to AM plants [77]. The modulation of gene expression encoding gibberellin (GA) biosynthetic enzymes, GA 20-oxidase and GA 2-oxidase, and the GA-signaling repressor DELLA in OM symbiosis was also found [78]. The latest research showed that exogenous GA treatment inhibits fungal colonization and decreases the percentage of seed germination during in vitro symbiotic germination of *D. officinale* [79]. Generally, GA is a crucial player in the germination of many plant seeds and AM symbiosis [80,81]. It is also most likely a key signal molecule for crosstalk between the seed germination pathway and mycorrhiza symbiosis during symbiotic orchid germination [79].

Nutrient transfer in OM symbiosis has been questioned for a long time. It is believed that OM fungi provide these plants with nutrient sources without an apparent reward, especially during the mycoheterotrophic stages [82]. Transcriptomic and proteomic approaches have been performed to clarify the nutrient transfer in OM (Table 2) [83,84]. Several *Serapias vomeracea* genes encode putative amino acid and oligopeptide transporters/permeases, which are strongly induced in symbiotic protocorms, suggesting that the transfer of organic nitrogen to the host plant in orchid mycorrhizae does exist [83]. The external mycelium of *Tulasnella calospora* surrounding *S. vomeracea* symbiotic protocorms showed specific lipid content [84]. Lipids have recently become an important topic in mycorrhizal research because such fungi are obligate biotrophs that cannot synthesize fatty acids; therefore, lipids are transferred from the plant [85,86]. Although most OMF are free-living decomposers that contain the genetic machinery for lipid biosynthesis, it is still unclear if the bidirectional transfer of nutrients occurs through intact pelotons or protocorms can acquire nutrients from the collapsed pelotons unilaterally. In a study using ultra-high spatial resolution secondary ion mass spectrometry (SIMS) with stable-isotope tracers to monitor carbon and nitrogen trafficking in peloton cells of the terrestrial orchid *Spiranthes sinensis*, it was strongly suggested that intact and senescent pelotons transfer carbon and nitrogen to host cells [87]. However, a similar study using high-resolution SIMS did not detect carbon movement across intact mycorrhizal interfaces between *Rhizanthella gardneri* and its mycorrhizal fungi up to 216 hours after ^13^CO_2_ labeling. Consequently, the necrotrophic uptake of nutrients derived from lysed pelotons was the dominant nutrient transfer method [88]. Since these two studies are different in many ways, such as plant species and symbiotic germination methods, further studies should be conducted to expound these discrepancies.

In situ or ex situ symbiotic germination is also a strong tool for understanding the orchid-fungus relationship (Table 2). For example, to compare mycorrhizal communities associated with the endangered orchid in Europe, *Liparis loeselii*, amplicon pyrosequencing was used to assess variations in OMF communities of protocorms across study areas. Fungal diversity varied among these sites, but these variations did not affect seed germination, indicating that the availability of fungal associates is not necessarily the determining factor that drives the distribution of endangered orchid species [89].

## 4. Future Perspective

Globally, orchid conservation remains a big challenge, especially to keep the plants established in natural habitats. To reintroduce orchids into natural habitats, a detailed understanding of mycorrhizal fungal associations is required [58]. Since in situ studies are complex and impractical [67], in vitro symbiotic germination studies will contribute to understanding the interaction between orchids and OMF in nature. Several studies also emphasized the importance of mycorrhizal interaction to plant quality during acclimatization, e.g., increase of leaf size, accelerated flowering time, and survival rate [10,13,39,90]. These parameters are important for a plant growing in its natural habitat due to dynamic conditions [91]. In addition, by using advanced technologies, as mentioned in previous sections, the inner mechanisms during OM formation can be elucidated and contributes to understanding the early establishment of OM. In conclusion, by conducting in vitro symbiotic germination, in vitro produced plants could be reintroduced into natural habitats with better quality to sustain their existence in nature.

## Figures and Tables

**Table 1:** Successful in vitro symbiotic germination attempts using orchid mycorrhizal fungi.

Orchid			Fungus			Reference
Subfamily	Tribe	Species	Family	Species	Isolate Source	
Vanilloideae	Pogonieae	*Cyrtosia septentrionalis* (Rchb.f.) Garay	Physalacriaceae	*Armillaria mellea* subsp. *nipponica* J.Y.Cha et Igarashi	basidiome of *A. mellea*	[33]
				*Armillaria gallica* Marxm.	tuber of *Gastrodia elata,* and roots of *C. septentrionalis*	
				*Armillaria tabescens* (Scop.) Emel	roots of *C. septentrionalis*	
			Meripilaceae	unknown species	protocorms from in situ seed baiting	
		*Pogoniopsis schenckii* Cogn.	Bionectriaceae	*Clonostachys* sp.	roots of *P. schenckii*	[20]
	Vanilleae	*Erythrorchis ochobiensis* (Hayata) Garay	Auriculariaceae	*Auricularia polytricha* (Mont.) Sacc.	a dead trunk of *Morus australis* in Japan, and an unknown source in Mexico (donated by the Institute for Fermentation, Osaka, Japan)	[40]
			Omphalotaceae	*Lentinula edodes* (Berk.) Pegler	fruiting body of *L. edodes*	[32]
		*Vanilla calyculata* Schltr.	Ceratobasidiaceae	*Ceratobasidium* sp.	roots of *Vanilla odorata*	[57]
		*Vanilla rivasii* Molineros	Ceratobasidiaceae	*Ceratobasidium* sp.	roots of *V. odorata* and *V. calyculata*	
			Tulasnellaceae	*Tulasnella* sp.	roots of *V. rivasii* and *V. odorata*	
Cypripedioideae	Cypripedieae	*Cypripedium macranthos* var. *rebunense* (Kudoh) Miyabe et Kudoh	Ceratobasidiaceae	*Rhizoctonia* sp., unknown anastomosis group	roots of *C. macranthos* var. *rebunense*	[12]
		*Paphiopedilum villosum* (Lindl.) Stein	Tulasnellaceae	*Tulasnella* sp.	roots of *P. villosum*	[11]
Orchidioideae	Chloraeeae	*Bipinnula fimbriata* (Poepp.) I.M.Johnst	Ceratobasidiaceae	*Ceratobasidium* sp.	roots of *B. fimbriata*	[6]
			Tulasnellaceae	*Tulasnella calospora* (Boud.) Juel	roots of *B. fimbriata*	
		*Gavilea australis* (Skottsb.) M.N.Correa	Ceratobasidiaceae	*Thanatephorus cucumeris* (A.B.Frank) Donk	roots of *Aa achalensis*	[59]
				*Ceratobasidium* sp.	roots of *G. lutea, G. australis,* and *Sacoila lanceolata*	
			Tulasnellaceae	*Tulasnella calospora* (Boud.) Juel	roots of *Codonorchis lessonii*	
	Cranichideae	*Aa achalensis* Schltr.	Magnaporthaceae	*Gaeumannomyces cylindrosporus* D.Hornby, Slope, Gutter. et Sivan	roots of *A. achalensis*	[43]
			Pezizaceae	uncultured *Pezizaceae*	roots of *A. achalensis*	
			Ceratobasidiaceae	*Thanatephorus cucumeris* (A.B.Frank) Donk	roots of *A. achalensis*	
		*Spiranthes novae-zelandiae* Hook.f.	Tulasnellaceae	*Tulasnella* sp.	roots of *S. novae-zelandiae*	[37]
		*Cynorkis purpurea* (Thouars) Kraenzl.	Ceratobasidiaceae	*Ceratobasidium* sp.	roots of *Aerangis* sp. and *C. purpurea*	[3]
			Serendipitaceae	*Serendipita* sp.	seedlings of *Polystachya concreta*	
			Tulasnellaceae	*Tulasnella* sp.	roots of *Angraecum magdalenae*, *C. purpurea*, and *Tylostigma* sp.	
		*Gymnadenia conopsea* (L.) R.Br.	Ceratobasidiaceae	*Ceratobasidium* sp. ^a^	roots of *G. conopsea*	[61]
		*Habenaria macroceratitis* Willd.	Ceratobasidiaceae	*Ceratorhiza* sp.	roots of *H. quinqueseta* and *H. macroceratitis*	[58]
			Tulasnellaceae	*Epulorhiza* sp.	roots of *Spiranthes brevilabris* and *Epidendrum conopseum*	
		*Habenaria quinqueseta* (Michx.) Eaton	Ceratobasidiaceae	*Ceratorhiza* sp.	roots of *H. quinqueseta*	[58]
		*Habenaria repens* Nutt.	Ceratobasidiaceae	*Ceratorhiza* sp.	roots of *H. quinqueseta* and *H. macroceratitis*	[58]
			Tulasnellaceae	*Epulorhiza* sp.	roots of *Spiranthes brevilabris* and *Epidendrum conopseum*	
		*Platanthera clavellata* (Michx.) Luer	Tulasnellaceae	*Epulorhiza inquilina* Currah, Zettler et McInnis	roots of *P. clavellata, P. cristata*, and *P. integrilabia*	[68]
				*Epulorhiza* sp.	roots of *P. ciliaris*	[68]
		*Platanthera leucophaea* (Nutt.) Lindl.	Ceratobasidiaceae	*Ceratorhiza* sp.	roots of *P. leucophaea*	[53]
			Tulasnellaceae	*Tulasnella calospora* (Boud.) Juel	roots of *in situ Anacamptis laxiflora*	
Epidendroideae	Arethuseae	*Arundina graminifolia* (D.Don) Hochr.	Tulasnellaceae	*Tulasnella* sp.	roots of *A. graminifolia* and seedlings from *ex situ* bating	[65]
		*Bletilla striata* (Thunb.) Rchb.f	Tulasnellaceae	*Tulasnella calospora* (Boud.) Juel	roots of *Diuris maculata, Thelymitra aristata, Paphiopedilum,* and unknown source	[55]
			Tulasnellaceae	*Tulasnella* sp.	roots of *Habenaria radiata*	[48]
			Serendipitaceae	*Serendipita vermifera* (Oberw.) P. Roberts	roots of *Thelymitra* sp.	[69]
		*Coelogyne nervosa* A.Rich	Tulasnellaceae	*Epulorhiza* sp.	roots of *Eulophia epidendreae*	[70]
	Epidendreae	*Cycnoches haagii* Barb.Rodr.	Tulasnellaceae	*Tulasnella* sp.	roots of *Cyrtopodium paludicola* and *Hoffmannseggella caulescens*	[13]
		*Cyrtopodium glutiniferum* Raddi	Tulasnellaceae	*Epulorhiza epiphytica* O.L. Pereira, Rollemb. et Kasuya	roots of *Epidendrum rigidum* and *Polystachya concreta*	[14]
				*Epulorhiza* sp.	roots of *Laelia milleri*	
		*Cyrtopodium paludicola* Hoehne	Tulasnellaceae	*Tulasnella* sp.	roots of *C. paludicola* and *Epidendrum secundum*	[60]
		*Cyrtopodium saintlegerianum* Rchb.f.	Tulasnellaceae	*Tulasnella* sp.	roots of *C. saintlegerianum*	[38]
		*Eulophia alta* (L.) Fawc.	Physalacriaceae	*Armillaria* sp.	roots of *E. alta*	[36]
		*Ionopsis utricularioides* (Sw.) Lindl	Ceratobasidiaceae	*Ceratobasidium* sp.	roots of *I. utricularioides*	[25,26]
		*Oeceoclades maculata* (Lindl.) Lindl.	Psathyrellaceae	*Psathyrella candolleana* (Fr.) Maire	roots of *O. maculata*	[31]
		*Oncidium sphacelatum* Lindl.	Ceratobasidiaceae	*Ceratobasidium* sp.	roots of *Oncidium donianum*	[44]
				*Thanatephorus* sp.	roots of *Rossioglossum grande*	[49]
		*Tolumnia variegata* (Sw.) Braem	Ceratobasidiaceae	*Ceratobasidium* sp.	roots of *T. variegata* and *I. utricularioides*	[26]
	Dendrobieae	*Dendrobium aphyllum* (Roxb.) C.E.C.Fisch	Tulasnellaceae	*Tulasnella* sp. ^a^	protocorms from in situ seed baiting	[8]
		*Dendrobium chrysanthum* Wall. ex. Lindl.	Ceratobasidiaceae	*Rhizoctonia oryzae-sativae* (Sawada) Mordue	unknown (Institute of Microbial Technology, India)	[56]
				*Rhizoctonia solani* Kühn	infected *Ipomoea batatas*	
	Epidendreae	*Encyclia tampensis* (Lindl.) Small	Tulasnellaceae	*Tulasnella irregularis* Warcup et P.H.B. Talbot	roots of *E. tampensis* seedling and mature plant	[67]
		*Epidendrum dalstromii* Dodson	Psathyrellaceae	*Coprinellus radians* (Desm.) Vilgalys	roots of various epiphytic orchids	[35]
		*Epidendrum nocturnum* Jacq.	Psathyrellaceae	*Coprinellus radians* (Desm.) Vilgalys	roots of various epiphytic orchids	[35]
			Tulasnellaceae	*Tulasnella calospora* (Boud.) Juel	roots of *Spiranthes brevilabris*	[53]
				*Tulasnella irregularis* Warcup et P.H.B. Talbot	roots of *E. tampensis* seedling and mature plant	[67]
		*Pleurothallis coriacardia* Rchb.f.	Hypocreaceae	*Ilyonectria* sp.	roots of *P. coriacardia*	[34]
			Psathyrellaceae	*Coprinellus* sp.	roots of *P. coriacardia*	
	Vandeae	*Aerangis ellisii* (B.S.Williams) Schltr.	Ceratobasidiaceae	*Ceratobasidium* sp.	protocorm of *A. ellisii*	[9]
		*Dendrophylax lindenii* (Lindl.) Benth. ex Rolfe	Ceratobasidiaceae	*Ceratobasidium* sp.	roots of *D. lindenii*	[52]
		*Vanda coerulea* Griff. ex Rolfe	Ceratobasidiaceae	*Rhizoctonia zeae* Voorhees	roots of *V. coerulea*	[16]
		*Vanda thwaitesii* Hook.f.	Ceratobasidiaceae	*Ceratobasidium* sp.	roots of *V. thwaitesii*	[39]

^a^ Seedling differentiation.

**Table 2:** Overview of current studies on orchid mycorrhizal symbiosis during the germination stage using “omics” and imaging technologies.

Orchid Species	Fungal Species	Germination	Technique/Method	Reference
*Anoectochilus roxburghii* (Wall.) Lindl.	Unpublished	in vitro	Transcriptome (Illumina HiSeq 4000)	[78]
*Bletilla striata* (D.Don) Hochr.	*Tulasnella* sp.	in vitro	Transcriptome (Illumina HiSeq 1500)	[77]
*Dendrobium officinale* Kimura et Migo	*Tulasnella* sp.	in vitro	Transcriptome (Illumina HiSeq 2000) and proteome (iTRAQ)	[76]
*Liparis loeselii* (L.) Rich	(Seed baiting)	in situ	Microbiome (454 amplicon pyrosequencing)	[89]
*Oncidium sphacelatum* Lindl.	*Ceratobasidium* sp.	in vitro	Proteome (2D LC–MS/MS coupled to iTRAQ)	[44]
*Rhizanthella gardneri* R.S.Rogers	(Seemingly) *Ceratobasidium* sp.	ex situ	Stable isotope imaging (NanoSIMS)	[88]
*Serapias vomeracea* (Burm.f) Briq.	*Tulasnella calospora* (Boud.) Juel	in vitro	Transcriptome (454 GS-FLX pyrosequencing)	[75]
*S. vomeracea* (Burm.f) Briq.	*T. calospora* (Boud.) Juel	in vitro	Transcriptome (Illumina HiSeq 2000)	[83]
*S. vomeracea* (Burm.f) Briq.	*T. calospora* (Boud.) Juel	in vitro	Proteome (UPLC-UHR-QqToF-MS)	[84]
*Spiranthes sinensis* (Pers.) Ames	*Ceratobasidium* sp.	in vitro	Stable isotope imaging (NanoSIMS)	[87]

Abbreviations: iTRAQ, Isobaric Tag for Relative and Absolute Quantitation; NanoSIMS, Nanoscale Secondary Ion Mass Spectrometry; UHR-QqToF, Ultra High Resolution-Qq-Time-Of-Flight; UPLC, Ultrahigh Performance Liquid Chromatography.
